# Supplementary material for: Imaging of super-fast dynamics and flow instabilities of superconducting vortices
Source: Nat Commun. 2017 Jul 20;8:85. doi: 10.1038/s41467-017-00089-3 (PMC5519736; doi:10.1038/s41467-017-00089-3)
Supplement: Supplementary file 1 — Supplementary Information [file 41467_2017_89_MOESM1_ESM.pdf]

File name: Supplementary Information
Description: Supplementary Figures, Supplementary Notes and Supplementary References

File name: Supplementary Movie 1
Description: Movie of $B_z(x, y)$ above the Pb microbridge measured by the SOT at $B_a = 2.7$ mT as the applied current I is increased from 0 to 21 mA and then decreased down to –20.8 mA. For $|I| < I_c$ stationary vortices are seen to be displaced as the current is incremented. For $I > I_c$ vortices penetrate through the left edge of the constriction at initial velocities of over 10 km/s and follow each other as they move across the microbridge. The vortex trajectories form a tree-like structure with a single stem that undergoes a series of bifurcations into branches. At higher current two stems are created forming two separate trees. Similar behavior is observed at negative currents. Frame size 12×12 μm^2, pixel size 40×40 nm^2, acquisition time 240 s/frame, at high currents the color scale is intentionally saturated at the edge for clarity. Selected frames from the movie are shown in the second row of Fig. 2.

File name: Supplementary Movie 2
Description: Movie of $B_z(x, y)$ above the Pb microbridge at $B_a = 4.2$ mT as the applied current I is increased from 12 mA up to 18.2 mA, which is the maximal sustainable current without a thermal quench. For $I < I_c$ stationary vortices are displaced as the current is incremented. For $I > I_c$ vortices penetrate through the left edge of the constriction forming a tree-like structure with a single stem that undergoes a series of bifurcations into branches. At higher current the number of stems increases to four forming four separate trees. Frame size 12×12 μm^2, pixel sixe 40×40 nm^2, acquisition time 240 s/frame.

File name: Supplementary Movie 3
Description: Movie of $B_z(x, y)$ above the Pb microbridge at $B_a = 5.4$ mT as the applied current I is increased from 12.2 mA up to the maximal sustainable current of 16.2 mA. For $I > I_c$ vortices penetrate through the left edge of the constriction forming a tree-like structure with a single stem that undergoes a series of bifurcations into branches. At the highest current four stems are present forming four trees. Frame size 12×12 μm^2, pixel sixe 40×40 nm^2, acquisition time 240 s/frame. Selected frames from the movie are shown in the third row in Fig. 2.

File name: Supplementary Movie 4
Description: Movie of $B_z(x, y)$ above the Pb microbridge at $B_a = 9.0$ mT as the applied current I is increased from 0 up to the maximal sustainable current of 11.8 mA. For $I > I_c$ vortices penetrate through the left edge of the constriction through six stems at the highest current. The vortices flow along parallel channels curved due to the construction geometry, apparently forming a moving Bragg glass state. Frame size 12×12 μm^2, pixel sixe 40×40 nm^2, acquisition time 240 s/frame. Selected frames from the movie are shown in the bottom row in Fig. 2.

File name: Supplementary Movie 5
Description: TDGL simulations of the time evolution of the Cooper-pair density $\Delta^2(x, y, t)$ showing vortex flow in superconducting microbridge in geometry equivalent to experimental conditions in $B_a = 2.7$ mT at applied current I slightly above I_c. The vortices penetrate at the narrowest point of the constriction and then spread out into channeled flow. The entire movie corresponds to $16 \cdot 10^3$ τ_{GL} or about 2.8 ns of experimental time. One second of the movie correspond to 10^3 τ_{GL} or 0.17 ns of experimental time.

File name: Supplementary Movie 6
Description: TDGL simulations of the vortex flow in superconducting microbridge as in Movie 5 showing a superposition of Cooper-pair density $\Delta^2(x, y, t)$ with the time averaged $\langle \Delta^2(x, y, t)\rangle_t$ accentuating the channel flow of vortices.

File name: Supplementary Movie 7
Description: TDGL simulations of the time evolution of the Cooper-pair density $\Delta^2(x, y, t)$ showing vortex flow in superconducting microbridge in geometry equivalent to experimental conditions in $B_a = 5.4$ mT and applied current I about twice I_c. The vortices penetrate at six entry points establishing different dynamic states. In the central channel the vortices flow at equivalent velocities of the order of 100 km/s significantly suppressing the time-averaged order parameter resulting in Abrikosov-Josephson dynamic vortex state. In the first channel to the left of the center the vortices are slipstreamed by the wake of the reduced order parameter of the preceding vortex. In the first channel to the right of the center the slipstreamed vortices dynamically transform into conventional Abrikosov vortices upon slow down after the channel bifurcation. In the more remote channels moving Abrikosov vortices are present. A frame from the movie is shown in Fig. 6a. The entire movie corresponds to 1500 τ_{GL} or about 260 ps of experimental time. One second of the movie correspond to 100 τ_{GL} or 17 ps of experimental time.

File name: Supplementary Movie 8
Description: TDGL simulations of the vortex flow in superconducting microbridge as in Movie 7 showing a superposition of Cooper-pair density $\Delta^2(x, y, t)$ with the time averaged $\langle \Delta^2(x, y, t)\rangle_t$ accentuating the channel flow of vortices.

File name: Peer Review File
Description:

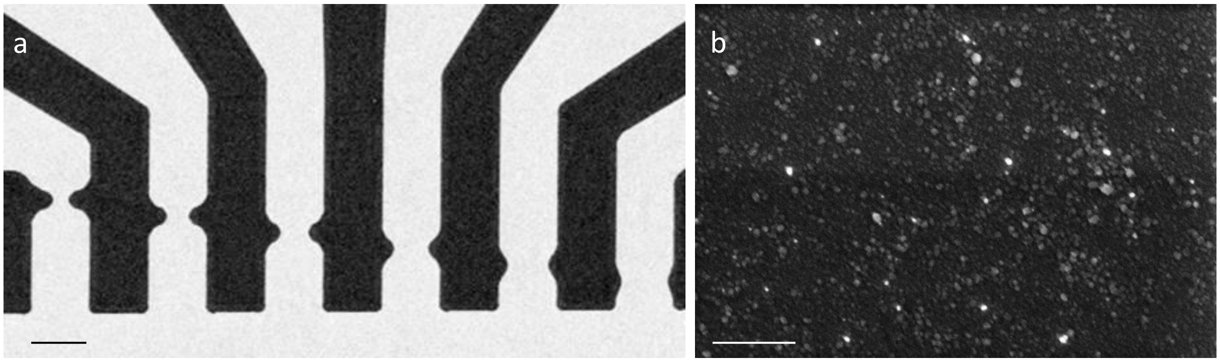

Supplementary Figure 1 | SEM images of the sample. (**a**) Large scale image showing the Pb film (light gray) on a Si substrate (dark). Six 10 µm wide microbridges with constrictions of different widths from 3 to 8 µm. Current can be applied independently to each of the bridges and voltage was measured at contacts outside the field of view. The scale bar is 10 µm (**b**) Higher magnification image of the surface of the Ge-capped Pb film showing grains with a typical diameter of a few tens of nm. The scale bar is 400 nm

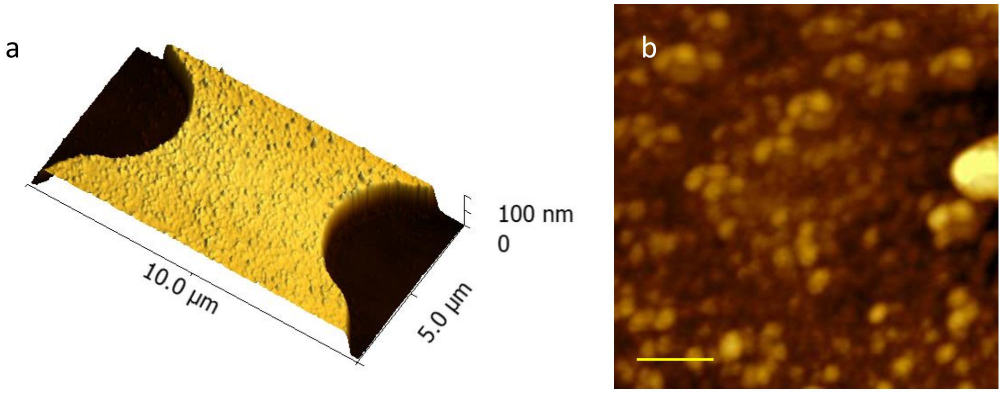

Supplementary Figure 2 | AFM topography of the Pb sample. (a) A 3D representation of the 10 µm-wide microbridge with 5.7 µm-wide constriction region. The film thickness is 82 nm; 75 nm of Pb capped by 7 nm of Ge. (**b**) Zoomed-in image of 1×1 μm^2 of the film surface revealing a granular structure with typical grain size of a few tens of nm. The color span is 18 nm. The scale bar is 200 nm

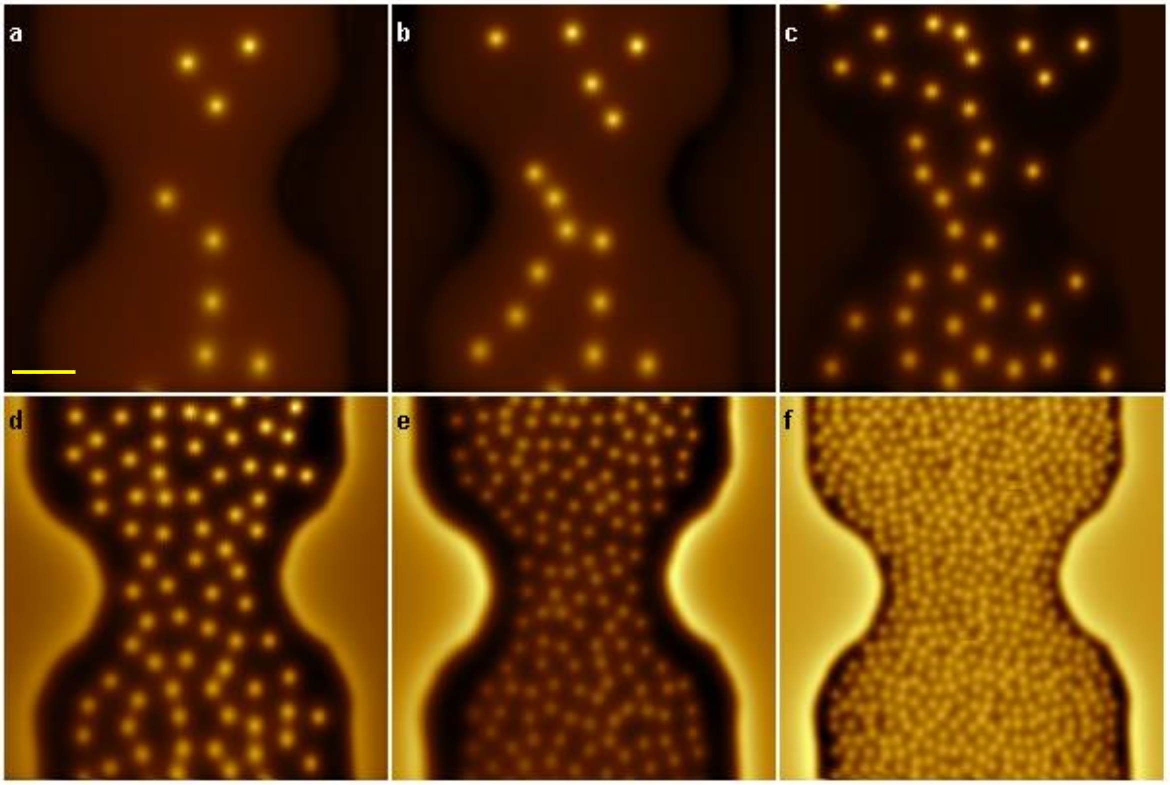

Supplementary Figure 3 | magnetic imaging of vortex configurations after field cooling. 12×12 μm^2 images showing vortices in the microbridge with the 5.7 μm-wide constriction after field cooling in fields of 0.3 (**a**), 0.6 (**b**), 1.5 (**c**), 2.7 (**d**), 5.4 (**e**), and 12 mT (**f**). The SOT scanning speed was 30 μm/s and the pixel size is 40x40 nm^2. The scale bar is 2 μm

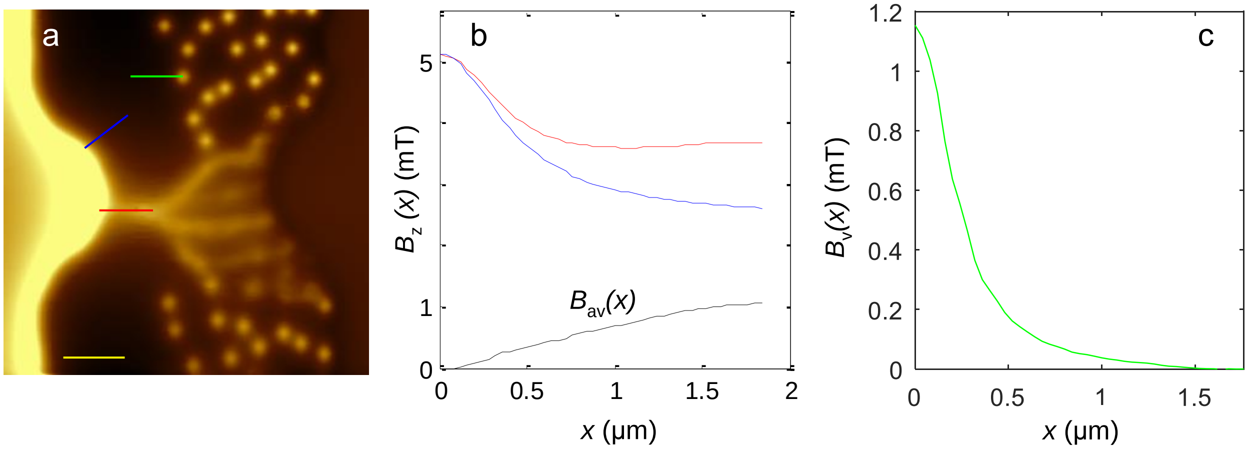

Supplementary Figure 4 | Analysis of field profiles along an isolated vortex and vortex stem. (**a**) $B_z(x,y)$ SOT image of the Pb microbridge at $B_a = 2.7$ mT and $I = 18.3$ mA. Scan area is 12×12 μm^2 and the field span is 3.1 mT. (**b**) $B_z(x)$ profiles along the lines marked in (a): Red – profile along the stem from the sample edge to the first bifurcation point x_b. Blue – background signal due to the Oersted field created by the edge currents. Black – $B_{av}(x)$ along the vortex stem after background subtraction. (**c**) Field profile along an isolated vortex $B_v(x)$ from the vortex center into the Meissner region (green line in (a)) after background subtraction. The scale bar is 2 μm

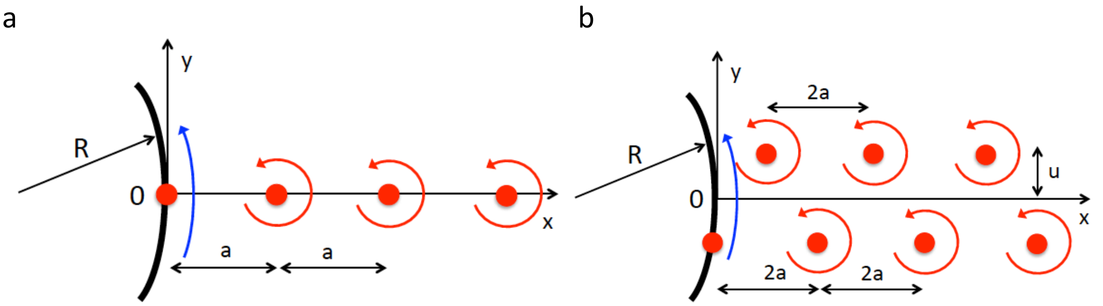

Supplementary Figure 5 | Current flow in vortex stems. (**a**) A single vortex chain of period a at the moment of penetration of a new vortex at $(x, y) = (0,0)$. The vortex and the Meissner currents are indicated by red and blue arrows, respectively. (**b**) Two vortex stems spaced by $l = 2u$ with anti-correlated vortex arrangement at the moment of penetration of a new vortex at $y = -u$.

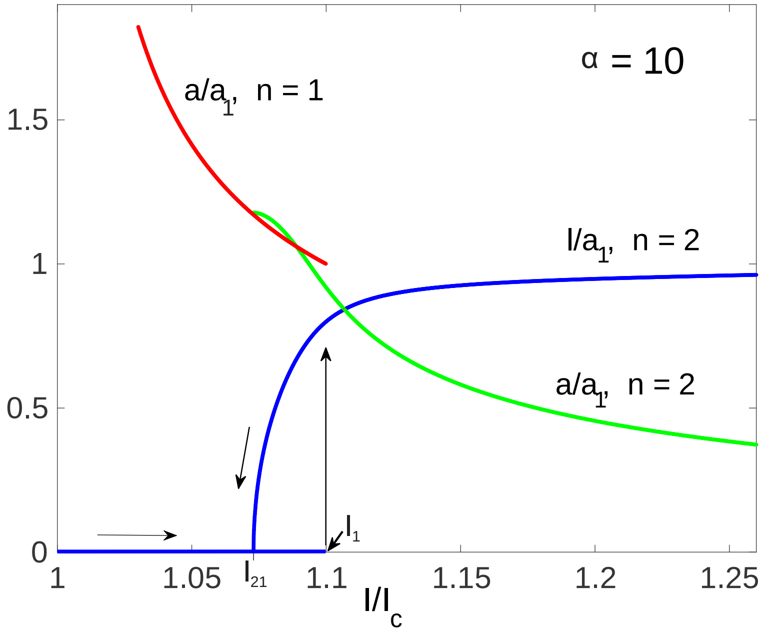

Supplementary Figure 6 | Inter-vortex distance a and inter-chain spacing l for single and double chain *vs.* applied current I. The curves $a(I)/a_1$ (red and green) and $l(I)/a_1$ (blue) were calculated from Supplementary Eqs. 34 and 35 for $\alpha = (R/\lambda)(d/5\sqrt{3}\xi)^{1/2} = 10$. The inter-vortex spacing $a(I)$ for a single chain ($n = 1$, red) decreases with I up to $a = a_1$ at $I = I_1$ above which the chain splits into two chains ($n = 2$, green). For $I > I_1$ the spacing between the chains $l(I)$ has a jump followed by a continuous increase. At $I = I_1$, $a(I)$ jumps from $n = 1$ curve (red) to $n = 2$ curve (green) and then decreases continuously until the next splitting transition. This chain-splitting transition is hysteretic: as I decreases, $l(I)$ decreases continuously and vanishes at $I = I_{21} < I_1$. For the presented case of $\alpha = 10$, $I_1 = 1.1I_c$ and $I_{21} = 1.07I_c$. For the parameters of our Pb bridge given in the text we attain $\alpha = 9$ resulting in $I_1 = 1.11I_c$ and $I_{21} = 1.08I_c$.

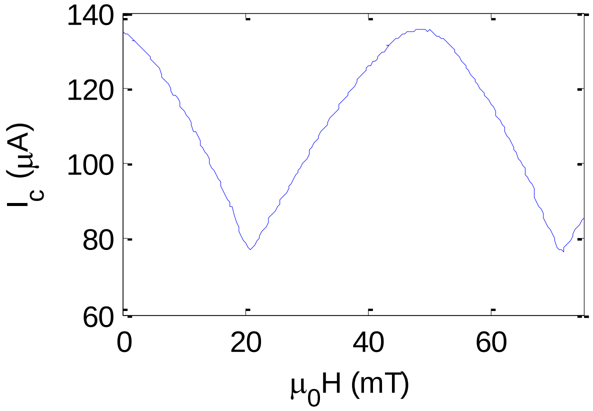

Supplementary Figure 7 | Quantum interference pattern of the SQUID-on-tip (SOT). The critical current I_c of the SOT used in this work *vs.* the applied field. The period of 50.7 mT of the quantum interference patter corresponds to an effective diameter of 228 nm of the SOT. The asymmetric structure of the SOT resulted in a slight asymmetry and a shift in the interference pattern giving rise to finite magnetic sensitivity of the SOT down to zero applied field.

Supplementary Note 1: TDGL description of moving vortex matter

As described in Methods, the dynamic evolution of the complex superconducting order parameter $\Psi(\boldsymbol{r},t)=\Delta(\boldsymbol{r},t)\cdot e^{-i\theta(\mathbf{r},t)}$ at temperatures close to the superconducting critical temperature T_c can be described by the generalized TDGL equation for a gapped dirty superconductor[1,2],

$$\frac{u}{\sqrt{1+\gamma^2|\Psi|^2}}\left(\frac{\partial}{\partial t}+i\varphi+\frac{\gamma^2}{2}\frac{\partial|\Psi|^2}{\partial t}\right)\Psi=(\boldsymbol{\nabla}-i\mathbf{A})^2\Psi+(1-|\Psi|^2)\Psi, \qquad (1)$$

coupled with the equation for electrostatic potential φ:

$$\boldsymbol{\nabla}(\sigma_n\boldsymbol{\nabla}\varphi)=\boldsymbol{\nabla}(Im\{\Psi^*(\boldsymbol{\nabla}-i\mathbf{A})\Psi\}). \qquad (2)$$

These equations are written in dimensionless form as defined in the Methods. In what follows, we discuss the parameters of the Pb sample under study, and some important estimates of the observable quantities in TDGL.

Related to the observed channeling of ultrafast vortices, we first discuss the conditions for appearance of a wake of suppressed order parameter behind a rapidly moving vortex core[3,4]. Following the approach from Refs. [2,3], we exclude the phase of the order parameter $\theta(\boldsymbol{r},t)$ from Supplementary Eqs. (1) and (2) and obtain the following equation for the magnitude of the order parameter $\Delta(r,t)$:

$$\tau_\Delta\partial_t\Delta=\Delta-\Delta^3+(\boldsymbol{\nabla}^2-\mathbf{Q}^2)\Delta. \qquad (3)$$

where $\mathbf{Q}=\boldsymbol{\nabla}\theta+\mathbf{A}$ is the gauge-invariant potential. The gap relaxation time τ_Δ is given as

$$\tau_\Delta=\tau_{\mathrm{GL}}u(1+\gamma^2\Delta^2)^{1/2}. \qquad (4)$$

Here, γ can be determined from the inelastic phonon-electron scattering time, $\tau_{\mathrm{in}}=2\hbar(sp_{\mathrm{F}})^2/7\pi\zeta(3)\lambda_{\mathrm{p}}(k_{\mathrm{B}}T)^3$ (s is the sound velocity, p_F the Fermi momentum, and λ_{p} the dimensionless electron-phonon coupling constant), as

$$\gamma=\left(\frac{64\sqrt{u\epsilon}}{7\pi^2\zeta(3)\lambda_{\mathrm{p}}}\right)\left(\frac{s}{v_{\mathrm{F}}}\right)^2\left(\frac{T_{\mathrm{c}}T_{\mathrm{F}}^2}{T^3}\right), \qquad (5)$$

where $\epsilon=1-T/T_{\mathrm{c}}$, $T_{\mathrm{F}}=p_{\mathrm{F}}v_{\mathrm{F}}/2k_{\mathrm{B}}$ is the Fermi temperature, and v_{F} is the Fermi velocity. Taking the available single-crystal values[5] for Pb, $T_{\mathrm{c}}^{(\mathrm{Pb})}=7.2$ K, $T_{\mathrm{F}}^{(\mathrm{Pb})}=1.1\times10^5$ K, $s^{(\mathrm{Pb})}=2$ km/s, $v_{\mathrm{F}}^{(\mathrm{Pb})}=1830$ km/s, $\lambda_{\mathrm{p}}^{(\mathrm{Pb})}=1.55$, we obtain $\gamma(T\approx T_{\mathrm{c}})\cong334\sqrt{\epsilon}$ and $\gamma(T=4.2\ \mathrm{K})\cong1085$. Such large values of γ indicate that the gap relaxation time τ_Δ is mostly determined by the electron-phonon scattering:

$$\tau_\Delta\cong\frac{\tau_{\mathrm{in}}}{\sqrt{\epsilon u}}=\frac{8\hbar}{7\pi\zeta(3)\lambda_{\mathrm{p}}k_{\mathrm{B}}T\sqrt{\epsilon u}}\left(\frac{sT_{\mathrm{F}}}{v_{\mathrm{F}}T}\right)^2. \qquad (6)$$

For the parameters listed above, Supplementary Eq. (6) yields $\tau_\Delta\approx0.2$ ns, and $\tau_{\mathrm{in}}\approx0.3$ ns at 4.2 K. The wake of suppressed order parameter behind the vortex core moving with the velocity v extends over the length Λ_{T} which can be obtained from Supplementary Eq. (3) as:

$$4\Lambda_{\mathrm{T}}=v\tau_\Delta+\sqrt{v^2\tau_\Delta^2+8\xi^2}. \qquad (7)$$

For $\xi=46$ nm, $\tau_\Delta=0.2$ ns, and $v=5$ km/s, we obtain $\Lambda_{\mathrm{T}}\cong0.5$ μm, so this short-range mechanism may not be sufficiently effective to align vortices typically spaced by ~ 1-2 μm in our Pb microbridge (in

relation to Fig. 4) but should become dominant at higher velocities, as is clearly observed in the dynamic vortex phases of Fig. 6.

Here we must emphasize that applicability of TDGL for gapped superconductors carries important limitations, which is why the simulations in the paper are conducted to reveal the new physics of ultrafast viscosity-dominated vortex dynamics rather than to claim that they reproduce the actual nonequilibrium properties of our specific sample. The derivation of TDGL assumes that: (1) the spatial variation of order parameter $\Psi(\boldsymbol{r},t)$ at temperatures $T \to T_c$ is slow over distances $r > \xi_0$, where ξ_0 is the BCS coherence length, and (2) the condition of quasi-equilibrium kinetics and the neglect of higher order temporal derivatives are valid if $\Psi(\boldsymbol{r},t)$ varies slowly over the inelastic phonon-electron scattering time[3], $\tau_{\rm in}$. For a moving vortex, the characteristic variation time is the velocity divided by the core size so that TDGL is applicable if $v \ll v_c \sim \xi/\tau_{\rm in}$. For $\xi = 46$ nm and $\tau_{in} \approx 0.3$ ns, one obtains $v_c \approx 0.2$ km/s at 4.2 K. Even though the exact parametrization of our Pb sample may differ from the one taken above, it is clear that using TDGL for calculations related to observed ultrafast-moving vortices is hardly justified, even though its results are illuminating and in line with the experimental observations. We therefore reiterate that our SOT experiments probe hitherto unexplored dynamics of very fast vortices, and at low temperatures, for which a suitable theory is yet to be developed. Such theory requires taking into account complicated nonequilibrium kinetics of superconductors coupled with strong pair-breaking effects in nonuniform distributions of superfluid density and current of moving vortex patterns.

Supplementary Note 2: Sample characterization

Supplementary Fig. 1a shows a SEM image of the sample. Six microbridges with constrictions of different widths (ranging from 3 to 8 µm) were patterned in the film, through which individual currents could be applied. The measurements presented here were performed on the third bridge from the right. The straight part of the bridge is 10 µm wide and the constriction is 5.7 µm wide and 5 µm long. All the transport measurements were performed in four probe configuration with voltage and current contacts outside the field of view of the SEM image. Since all the leads are significantly wider than the constriction, the current density in them is well below J_c and hence no vortex flow is induced. As a result, the measured finite V arises only from vortex flow in the constriction.

The zoomed-in SEM image in Supplementary Fig. 1b reveals a granular structure typical of metallic surfaces with grain size on the order of a few tens of nm. An AFM topography image of a constricted bridge is shown in 3D representation in Supplementary Fig. 2a. The overall thickness of the film was measured to be 82 nm which consists of a 75 nm-thick Pb film and a 7 nm-thick Ge capping layer. Supplementary Figure 2b is a high resolution 1×1 µm^2 scan of the surface showing granular structure with a typical grain size of a few tens of nm, consistent with the SEM images.

Supplementary Note 3: Extraction of the vortex velocity along a channel

The time-averaged field $B_{av}(x)$ along a chain of vortices separated by $a(x)$ and moving at velocity $v(x) = fa(x)$ is given by $B_{av}(x) = \int_{-\infty}^{\infty} B_v(u)du/a(x) = f\int_{-\infty}^{\infty} B_v(u)du/v(x)$ (see below), where $B_v(u)$ is the magnetic field profile of an individual vortex. By measuring $B_{av}(x)$ along the stem and $B_v(x)$ across an isolated stationary vortex, we can thus attain $a(x)$ along a single stem (Fig. 4a) and combining it with the penetration rate f derived from simultaneous transport measurements (Fig. 3c), we obtain the corresponding vortex velocity $v(x)$ (Fig. 4b).

Supplementary Fig. 4a shows the measured $B_z(x,y)$ at $B_a = 2.7$ mT and $I = 18.3$ mA with a single vortex stem. The field profile along a stem is shown in Supplementary Figure 4b (red). Since the SOT image is acquired at a height of $h \sim 200$ nm above the film, it contains also the B_z component of the Oersted field of the currents flowing near the edge of the strip. We subtract this contribution using the B_z profile in the

nearby Meissner region (blue), attaining $B_{av}(x)$, shown by the black curve in Supplementary Fig. 4b. Similarly, the field profile of a single vortex $B_v(x)$ is obtained along the green line extending from the center of the vortex into the Meissner region, as shown in Supplementary Fig. 4c. We then integrate numerically the field of a single vortex $\int B_v(u)\,\mathrm{d}u$ and repeat this procedure over several vortices in various images with observed variations of a few percent. The corresponding $a(x)$ is then derived from the ratio of $B_{av}(x)$ and $\int B_v(u)\,\mathrm{d}u$, as presented in Fig. 4 in the main text.

We note here some aspects pertaining to the validity of the above procedure.

a) The measured field profile across the stationary vortex in Supplementary Fig. 4c shows FWHM width of about 0.5 μm which is much larger than $\xi = 46$ nm and $\lambda = 96$ nm. This wide profile is a result of the magnetic size of the vortex given by the Pearl penetration depth $\Lambda = 2\lambda^2/d \cong 245$ nm further broadened by the SOT diameter of 228 nm and the SOT scanning height of about 200 nm. Under these conditions the vortices are seen by SOT as a point magnetic monopole with flux ϕ_0. Hence the SOT images are insensitive to the elongation of the vortex core along the direction of motion, unless the core becomes longer than Λ which is not the case in the experiment as discussed in the main text.

b) The derivation of vortex velocity does not include details of the vortex profile $B_v(x)$ itself but only the line integral of it. Since the areal integral of $B_v(x,y)$ equals ϕ_0 regardless of the vortex structure, the line integral is significantly less sensitive to the microscopic structure of the vortex than $B_v(x)$ itself.

c) The above-described procedure is not valid very close to the edge of the constriction due to the image antivortex imposed by the boundary conditions. Consider the perpendicular component of the magnetic field $B_v(x,y,h)$ produced by the Pearl vortex spaced by u from the film edge[6] at $x = 0$. To simplify the expressions we take the height above the film to be $h \gg \lambda^2/d$ resulting in

$$B_v(x,y,h) = \frac{\mu_0\phi_0 h}{2\pi[(x-u)^2 + y^2 + h^2]^{\frac{3}{2}}} - \frac{\mu_0\phi_0 h}{2\pi[(x+u)^2 + y^2 + h^2]^{\frac{3}{2}}}, \qquad (8)$$

where the second term on the right comes from the vortex image. Let vortices appear periodically at the edge and move rapidly along x so that the SOT signal $B_{av}(x,y)$ is proportional to $B_v(x,y,h)$ averaged over the instantaneous positions of vortices with the probability-density $\propto 1/v(x)$

$$B_{av}(x,y) \propto \int_0^\infty \left\{ \frac{h}{[(x-u)^2 + y^2 + h^2]^{\frac{3}{2}}} - \frac{h}{[(x+u)^2 + y^2 + h^2]^{\frac{3}{2}}} \right\} \frac{du}{v(u)}. \qquad (9)$$

For a constant $v(u)$, integration of Supplementary Eq. (9) yields

$$B_{av}(x,y) \propto \frac{xh}{v(y^2 + h^2)\sqrt{x^2 + y^2 + h^2}}. \qquad (10)$$

The vanishing SOT signal at the edge $x = 0$ results from the extinguishing of the vortex field by its image. Along the center of the channel ($y = 0$) the contribution of the vortex image becomes negligible for $x > h$. For this reason we ignored the experimental data at $x < 0.5$ μm.

If $v(x)$ varies slowly over the length $\sim h$, the function $v(u)$ in Supplementary Eq. (9) can be expanded around the point $x = u$, $y = 0$ where the integrand is peaked, and $v^{-1}(u) \approx v^{-1}(x) - (x-u)v'(x)/v^2(x)$, where the prime denotes the x-derivative. Then integration in Supplementary Eq. (9) gives the following relation:

$$B_{av}(x) \propto \frac{x}{v(x)h\sqrt{x^2+h^2}} - \frac{xhv'(x)}{v^2(x)\sqrt{x^2+h^2}\left(x+\sqrt{x^2+h^2}\right)} \qquad (11)$$

For $x > h$ and $v(x)$ varying slowly over the length $\sim h$, the second term on the right of Supplementary Eq. (11) is negligible and it results in the direct relation $B_{av}(x) = f\int_{-\infty}^{\infty} B_v(u)du/v(x)$ used in our data analysis where $B_v(u)$ is the field profile of an isolated vortex.

d) In addition to the derivation of the local vortex velocity presented in Fig. 4, we performed an independent evaluation of the average vortex velocity in the stem which does not rely on $B_v(x)$. It is based only on the measurement of the voltage drop on the bridge and on the average vortex distance determined by the applied field. The obtained average velocity of 15 km/s is consistent with the derived local velocities in Figs. 4b and 4c.

Supplementary Note 4: Thermal confinement of vortex chains and buckling instability

Long-range confinement of moving vortices can result from the Joule heating they produce. Consider the equation for the steady-state temperature $\theta(x,y) = T(x,y) - T_0$ relative to the bath temperature T_0 due to heating by a chain of vortices spaced by a and moving with the velocity v:

$$\kappa\nabla^2\theta + \eta v^2 \sum_n f(x - vt - na, y) - \frac{K}{d}\theta = 0 \qquad (12)$$

where κ is the thermal conductivity, η is the viscous drag coefficient and K is the Kapitza thermal conductance between the film and the substrate[7]. The second term describes the power dissipated by a moving vortex chain and the last term describes heat transfer from the film to a substrate. Supplementary Equation (12) is written in the coordinate frame moving with vortices along the x-axis and describes $\theta(x,y)$ averaged over the washboard frequency v/a (similar equation can be used to describe electron heating[3,8]). Here the dynamic term $Cv\partial_x\theta$ proportional to the specific heat C is neglected, assuming that $v < v_T \sim \kappa/Ca$. For typical values[9] of $C = 2$ kJ/m^3 K, $\kappa = 200$ W/mK and $a = 1$ μm, we have $v_T = 100$ km/s, well above the velocities measured in our SOT experiments. We also introduce the normalized weight function $f(x,y)$ which provides a smooth vortex core cutoff in the London model:

$$f(x,y) = \frac{1}{4\pi\xi^2} exp\left(-\frac{x^2+y^2}{4\xi^2}\right) \qquad (13)$$

For weak heating, $\theta(x,y) \ll T_0$, temperature dependencies of κ and K are negligible, and the solution of Supplementary Eq. (12) is given by the Fourier transform

$$\theta(x,y) = \frac{\eta v^2}{\kappa a}\sum_G \int_{-\infty}^{\infty} \frac{\exp[-(q^2+G^2)\xi^2 + iqy + iGx]dq}{2\pi(q^2+G^2+L_T^{-2})}, \qquad (14)$$

where $G = 2\pi n/a,\ n = 0, \pm 1, \pm 2, \ldots$, and the thermal length L_T defines a spatial scale of temperature variation along the film

$$L_T = (d\kappa/K)^{1/2}. \qquad (15)$$

For typical values[9] of $\kappa =$ (200-300) W/mK, $K =$ 10-50 kW/m^{2}K at 4.2 K and $d = 50$ nm, Supplementary Eq. (15) yields $L_T =$ 10-30 μm, much greater than the vortex spacing, so that variation of $\theta(x,y)$ along the vortex chain is negligible and the main contribution in Supplementary Eq. (14) comes from the term with $G = 0$ in the sum. In this case, calculation of the integral in Supplementary Eq. (14) and $|y| \gg \xi$ gives the temperature distribution across the vortex chain:

$$\theta(y) = \frac{\eta L_T v^2}{2\kappa a} exp\left(-\frac{|y|}{L_T}\right). \quad (16)$$

At $|y| \sim \xi$, the finite size of the vortex core in Supplementary Eq. (14) becomes important as it rounds the cusp in Supplementary Eq. (16) at $y = 0$, resulting in a finite curvature $\theta''(y)$ inversely proportional to ξ:

$$\theta''(0) = -\frac{\eta v^2}{2\sqrt{\pi}\xi a\kappa}. \quad (17)$$

Unlike $\theta(y)$ at $|y| \gg \xi$ in Supplementary Eq. (16), $\theta''(0)$ in Eq. (4.6) is independent of the Kapitza conductance K and thus of details of heat transfer from the film to the substrate.

To estimate the maximum heating $\theta_m = \theta(0)$ in the moving vortex chain, we use Supplementary Eqs. (15) and (16) along with the power balance, $\eta v^2/a = IV/dw$ where V is the constant voltage on the chain, I is the total current and w is the film width. Hence,

$$\theta_m = \frac{IV}{2w\sqrt{dK\kappa}}. \quad (18)$$

Taking here typical values of $I = 20$ mA, $V = 30$ μV, $d = 50$ nm, $w = 5$ μm, $K = 10$ kW/m^{2}K and $\kappa = 200$ W/mK for our Pb microbridge, yields rather weak heating $\theta_{\rm m} = 0.2$ K, which can hardly cause thermomagnetic branching instabilities[10,11].

However, even such weak heating can align vortices in a chain because the long-range temperature field extends over distances $L_{\rm T}$ much greater than the inter-vortex spacing. As a result, a moving vortex chain produces a self-consistent "temperature well" which prevents buckling instability of repulsive vortices. Indeed, if a single vortex is shifted across the chain by u, it experiences the restoring thermal forces $f_{\rm T} = -s^*\nabla\theta$, where $s^*(T)$ is the transport entropy per unit vortex length[12]. Using $\theta(y)$, calculated above, we obtain $f_{\rm T} = -s^*\theta''(0)u$, where $\theta''(0)$ is given by Supplementary Eq. (17). Hence,

$$f_{\rm T} = \frac{\eta v^2 s^* u}{2\sqrt{\pi}\xi a\kappa} \quad (19)$$

The thermally-induced confinement force f_T increases as vortices move faster and get closer, so that the depth of the thermal well increases with the dissipated power IV. Yet $f_{\rm T}$ in Supplementary Eq. (19) is independent of the thermal Kapitza conductance between the film and the substrate because $L_{\rm T} \gg \xi$.

Shifting a single vortex by u across the chain also causes a Lorentz force $f_{\rm m}(u)$ from other vortices that pushes the vortex further away from the chain. Using the repulsive force $F_{\rm m} = (\phi_0/2\pi r)^2$ between two vortices separated by $r > \lambda^2/d$ in a thin film[13], we calculate the sum of the y-components of the inter-vortex interaction forces per unit length $f_{\rm m} = F_{\rm m}/d$ acting on a vortex shifted by $u < a$:

$$f_{\rm m} = \frac{\phi_0^2}{2\pi^2 d}\sum_{n=1}^{\infty}\frac{u}{(a^2n^2 + u^2)^{3/2}} \cong \frac{\phi_0^2\zeta(3)u}{2\pi^2 da^3}. \quad (20)$$

The vortex chain is stable with respect to buckling distortions if $f_{\rm T} > f_{\rm m}$. It is convenient to express the vortex velocity in terms of the voltage $V = v\phi_0/ca$ and to write the stability condition $f_{\rm T} > f_{\rm m}$ in the form

$$a > a_{\rm c} = \left[\frac{\zeta(3)\kappa\phi_0^4\xi}{\pi^{3/2}\eta V^2 c^2 ds^*}\right]^{1/4}. \quad (21)$$

This criterion shows that the branching instability of a vortex chain occurs in the region of the film where the current density decreases so that vortices slow down and the spacing $a(x)$ decreases below a_{c}. For a rough estimate of a_{c}, we use[12] $\eta = \phi_0^2/2\pi\xi^2\rho_n c^2$ and $s^*\sim(\phi_0/4\pi\lambda)^2 T/T_{\mathrm{c}}^2$ where ρ_{n} is the normal state resistivity:

$$a_{\mathrm{c}} \sim 2\sqrt{\lambda\xi}\left[\frac{\kappa\rho_n T_c^2\xi}{V^2 T d}\right]^{1/4} \sim 2\sqrt{\frac{\lambda\xi T_c}{V}}\left(\frac{L_N\xi}{d}\right)^{1/4}. \qquad (22)$$

In the last expression, a_{c} was further simplified by using the Wiedemann-Frantz law $\kappa\rho_{\mathrm{n}} = TL_{\mathrm{N}}$ with the Lorentz number $L_{\mathrm{N}} = (\pi k_B/e)^2/3 = 2.44\cdot10^{-8}$ W·Ω/K^2. In this case, a_{c} becomes independent of the thermal materials parameters. For $V = 25$ μV, Supplementary Eq. (22) gives $a_c\sim 0.8$ μm, consistent with typical inter-vortex spacing in the branching vortex chains revealed by SOT. Supplementary Equation (22) also predicts that $a_{\mathrm{c}} \propto V^{-1/2}$. a_{c} increases as vortices slow down and become closer to each other, thus reducing the depth of the thermal well and increasing the buckling effect of inter-vortex repulsion. The dashed line in Fig. 4d is a plot of $a_c(V)$ given by Supplementary Eq. (22) using our Pb film parameters with no additional fitting parameters, showing good qualitative agreement.

Supplementary Note 5: Effects of disorder on premature branching

The above consideration addressed the buckling instability of a uniformly moving vortex chain with respect to infinitesimal bending distortions. In our experimental situation of $J \gg J_{\mathrm{c}}$ the effect of pinning on the viscosity-dominated vortex dynamics is weak and the channel bifurcation occurs primarily as the separation between the slowing down vortices drops below a_{c} as described above. For the sake of completeness, we evaluate here the contribution that disorder may have on distorting the thermal alignment of vortices in a chain, possibly causing premature branching instabilities even if the stability condition $a > a_{\mathrm{c}}$ for a fully aligned channel is satisfied.

The correlation function of pinning-induced transverse vortex displacements $\langle u_y(x)u_y(0)\rangle \propto x$ increases with the distance along the chain, which indicates buckling instability for a vortex chain longer than the critical length x_c. The increase of $\langle u_y(x)u_y(0)\rangle$ with x, similar to the well-known result of the collective pinning theory[13], can be obtained from the dynamic equation for $u_y(x,y,t)$:

$$\eta\partial_t u_y = -\partial_y U(x-u_x, y-u_y) \qquad (23)$$

where $U(x,y)$ is a random pinning potential with zero mean $\langle U(x,y)\rangle = 0$. For rapidly moving vortices at $J \gg J_{\mathrm{c}}$ where pinning induced potential is strongly suppressed, disorder can be treated perturbatively, replacing in the first approximation $U(x-u_x, y-u_y) = U(x-vt, y)$, where v is the free flux flow velocity[13]. Then the correlation function becomes

$$\eta^2\langle u_y(t)u_y(t')\rangle = \lim_{\substack{y\to0,\\ y'\to0}} \frac{\partial^2}{\partial y\partial y'}\int_0^t dt_1\int_0^{t'} dt_2\langle U(vt_1,y)U(vt_2,y')\rangle, \qquad (24)$$

where $x = vt$. We illustrate the qualitative features of $\langle u_y(x)u_y(0)\rangle$ for the Gaussian pinning correlation function:

$$\langle U(x,y)U(x',y')\rangle = U_0^2\mathrm{e}^{-R^2/2l^2}, \qquad (25)$$

where $R = |\boldsymbol{r}-\boldsymbol{r}'|$ and U_0 quantify the strength of pinning, while the correlation length l depends on the interaction radius of pinning centers and their spatial correlation. Substituting Supplementary Eq. (25) and integrating yields

$$\langle u_y(t)u_y(t')\rangle = \frac{\sqrt{2\pi}U_0^2}{\eta^2 v^2 l}x. \qquad (26)$$

Supplementary Equation (26) shows that, as vortices move along the chain, their pinning-induced transverse displacements $\langle u_y^2\rangle^{1/2} \propto x^{1/2}$ increase with x, particularly as vortices slow down. Pinning thus provides a destabilizing mechanism which may cause premature bifurcation of vortex chains as they propagate into the film. A similar effect was observed in numerical simulations of thermomagnetic flux avalanches in thin films where random inhomogeneities of pinning can greatly increase dendritic branching of propagating flux filaments[10].

Supplementary Note 6: Dynamics of stem nucleation in London model

We address the mechanisms that lead to nucleation of additional vortex stems using the London theory. Suppose that thermal or other mechanisms of confinement provide effective alignment of vortices in a stem formed at the edge in the narrowest part of the bridge (see Fig. 2g). Let us now evaluate the spacing $a(I)$ between vortices in the stem, and the conditions under which such a stem becomes unstable with respect to nucleation of an additional stem. In the Meissner state of a wide thin-film bridge carrying transport current I in a perpendicular magnetic field H, the maximum current density $J_{\mathrm{m}}(y)$ flows at the edge at which the magnetization currents are parallel to transport current[14]:

$$J_{\mathrm{m}}(y) \cong \frac{4I + cHw(y)}{4\pi d^{3/2} w^{1/2}(y)} \qquad (27)$$

Here we assume that the width of the microbridge $w(y)$ varies slowly over the Pearl length $\Lambda = 2\lambda^2/d$, and the width of the narrowest part of the bridge can be approximated by

$$w(y) = \left(1 + \tfrac{y^2}{R^2}\right) w_0, \qquad (28)$$

where $R \gg \Lambda$ is a characteristic curvature radius of the constriction (about 2 μm for our Pb microbridge). The non-dissipative Meissner state persists as long as $I < I_{\mathrm{c}}$, where the critical current I_{c} is defined by the condition that $J_{\mathrm{m}}(0)$ at the edge of the narrowest part of the bridge reaches the depairing current density J_{d} at which the barrier for penetration of vortices vanishes:

$$I_{\mathrm{c}} = \left(1 - \frac{H}{H_0}\right) I_0 \qquad (29)$$

$$I_0 \sim \pi d^{3/2} w_0^{1/2} J_d, \qquad H_0 \sim 4I_0/cw_0.$$

At $I > I_{\mathrm{c}}$ and $H = 0$, the Meissner current density exceeds J_{d} in a segment $-L_{\mathrm{m}} < y < L_{\mathrm{m}}$ of length $2L_m = 2R(2(I/I_0 - 1))^{1/2}$ along the rim. As a result, a chain of vortices penetrates at $y = 0$ to produce current counterflow, which reduces the edge value of $J(y)$ below J_{d}. Consider for simplicity a periodic chain with a constant intervortex spacing $a(I)$ determined by the condition that $J_m(0)$ reaches J_{d} as the last vortex in the chain closest to the edge is at the distance $a(I)$ from the edge, so a new vortex penetrates (Supplementary Figure 5).

A chain of Pearl vortices produces the following current density along the rim:

$$J_v(y) = \frac{c\phi_0}{2\pi^2} \sum_{n=1}^{\infty} \frac{na}{(a^2n^2 + y^2)^{3/2}}\,. \qquad (30)$$

The intervortex distance $a(I,H)$ is defined by the condition $J_{\mathrm{m}}(0) - J_v(0) = J_{\mathrm{d}}$ which yields:

$$a(I,H) = \pi\lambda\left(\frac{\xi\sqrt{3}}{d}\right)^{1/2}\left[\frac{I_0}{I - I_{\mathrm{c}}(H)}\right]^{1/2}, \qquad (31)$$

where we used the GL expression, $J_{\mathrm{d}} = c\phi_0/12\sqrt{3}\pi^2\lambda^2\xi$, assuming no defects at the edge.

A single vortex chain exists only in a certain range of currents $I_{\mathrm{c}} < I < I_1$ where the net edge current density $J_s(y) = J_{\mathrm{m}}(y) - J_{\mathrm{v}}(y)$ is below J_d everywhere along the rim except the entry point $y = 0$ where $J_s(y)$ is maximum and equals J_{d}. As I increases, the region $-L_{\mathrm{m}}(I) < y < L_{\mathrm{m}}(I)$ expands, so the counterflow of a single vortex chain can no longer sustain $J_s(y)$ below J_d everywhere along the rim. At $I = I_1$, the second derivative $\partial_{yy}J_{\mathrm{s}}(y)$ at $y = 0$ changes sign and the function $J_{\mathrm{s}}(y)$ has two maxima at two symmetric points $y = \pm u$ and $y = 0$ becomes a local minimum of $J_{\mathrm{s}}(y)$. From the condition, $\partial_{yy}J_v(y) = \partial_{yy}J_{\mathrm{m}}(y)$ at $y = 0$, we calculate I_1 and the spacing $a_1 = a(I_1)$ at $H = 0$:

$$I_1 = \left[1 + \frac{\lambda}{R}\left(\frac{5\sqrt{3}\xi}{d}\right)^{1/2}\right] I_0\,, \qquad (32)$$

$$a_1 = \pi\left(\frac{\sqrt{3}\xi}{5d}\right)^{1/4}\sqrt{\lambda R}\,. \qquad (33)$$

For $R = 1.5$ μm and $\lambda = 96$ nm, Supplementary Eqs. (32) and (33) yield $a_1 \approx 0.9$ μm and I_1 about 15% higher than I_{c}, qualitatively consistent with the experimental data shown in Fig. 3b.

At $I > I_1$, instead of a single stem, two stems are formed at $y = u$ and $y = -u$ with anti-phase vortex arrangement as shown in Supplementary Figure 5. Such a structure gives $J_{\mathrm{s}}(y) < J_{\mathrm{d}}$ everywhere along the rim except at the points $y = \pm u$ where new vortices enter. The next vortex enters at $y = -u$, so the condition $J_{\mathrm{s}}(-u) = J_{\mathrm{d}}$ and $\partial_y J_{\mathrm{s}}(-u) = 0$ give two coupled equations for the period $a(I)$ within each stem and the inter-stem spacing $2u(I)$ derived using Supplementary Eq. (30):

$$J_{\mathrm{d}} = \frac{I}{\pi d^{3/2} w_0^{1/2}}\left(1 - \frac{u^2}{2R^2}\right) - \frac{c\phi_0}{48da^2} - \frac{c\phi_0}{2\pi^2 d}\sum_{n=1}^{\infty}\frac{(2n-1)a}{[a^2(2n-1)^2 + 4u^2]^{3/2}} \qquad (34)$$

$$\frac{I}{\pi d^{3/2} w_0^{1/2} R^2} = \frac{3c\phi_0}{\pi^2 d}\sum_{n=1}^{\infty}\frac{(2n-1)}{[a^2(2n-1)^2 + 4u^2]^{5/2}} \qquad (35)$$

Shown in Supplementary Figure 6 are the results of numerical simulations of Eqs. (34, 35) for $\alpha = (R/\lambda)(d/5\sqrt{3}\xi)^{1/2} = 10$. The red line shows the vortex spacing $a(I)$ for a single stem, the green line shows the intra-stem vortex spacing $a(I)$ when two stems are present, and the blue line shows the inter-stem spacing $l(I)$. The essential feature of these results is a hysteretic single-to-double stem transition at $I = I_1$, when l jumps from 0 to $0.7a_1$, while $a(I)$ drops from a_1 to $0.88a_1$. The hysteresis occurs in the range of currents $I_{21} < I < I_1$ where $I_{21} = [1 + 4(\xi/2\sqrt{3}d)^{1/2}\lambda/R]I_{\mathrm{c}}$. As I increases further, a cascade of splitting transitions to four and more stems occurs.

Thus, we have derived above the criterion of the absolute instability of the vortex chain located at the point of a minimum of the width $w(y)$ (at $y = 0$). According to this criterion, at the current I_1, the maximum in the edge current density $J_s(y) = J_{\mathrm{m}}(y) - J_v(y)$ at $y = 0$ evolves into a minimum of this

function, and the vortex stem splits into the two equivalent stems shifted from $y = 0$. This splitting gives rise to a two-fold decrease in the vortex penetration rate per stem, as observed in Fig. 3c.

In the above scenario, the function $J_s(y) = J_m(y) - J_v(y)$ has a global maximum at $y = 0$ if $I < I_1$. However, due to irregularities of the sample edges, $J_s(y)$ can have an additional local maximum at some point $y_0 \neq 0$ because $J_m(y)$ and $J_v(y)$ have different dependences on y. Consider the following simple example: an infinite strip with a small constriction in the form of two semicircles of radius $r \ll w$ centered at $y = 0$ and $x = 0, w$. If the critical current without the constriction is I_c, the critical current of the strip with the constriction is reduced to $\tilde{I}_c = I_c(1 - 2r/w)^{1/2} \cong (1 - r/w)I_c$. According to Supplementary Eq. (32), a second stem at the constriction appears at the current I_1, which can be larger than I_c if $r^2 < w\lambda\left(5\sqrt{3}\xi/d\right)^{1/2}$. But at large y, the current density $J_v(y) \to 0$, and the edge current density $J_s(y) \approx J_m(y)$, which reaches the depairing current density J_d at the current $I = I_c < I_1$. In this case $y_0 \to \infty$, and the second stem will appear $I = I_c < I_1$ far away from the constriction. If there is another small constriction or edge irregularity along the strip the second stem will be nucleated there rather than at infinity, so that the initial stem does not change its position upon appearance of the second stem.

The intervortex distances in the stems are now defined by the equations:

$$J_m(0) - J_v(0) - J_{v1}(0) = J_d \qquad (36)$$

$$J_m(y_0) - J_v(y_0) - J_{v1}(y_0) = J_d, \qquad (37)$$

where $J_{v1}(y)$ is the edge current density generated by the vortices in the second stem. If y_0 is sufficiently large, we can neglect $J_{v1}(0)$ in Supplementary Eq. (36) and $J_v(y_0)$ in Supplementary Eq. (17) so that the vortex penetration process in the two stems is decoupled, each having its own penetration rate and independent $V - I$ characteristic, and the overall characteristic of the strip will be given by the sum of the two. Since nucleation of an independent stem is associated with a jump in V at I_c, the appearance of the second stem at I_1 will be accompanied by a jump in the total voltage of the strip comparable to the jump at I_c. This consideration shows that, if the edge of the bridge constriction has small protuberances of radius r << R, the first stem does not split into two stems which are symmetric with respect to y = 0. Instead, the first stem may remain near the narrowest point of the bridge at y = 0, whereas the second stem appears at a point where the nearest edge protuberance is.

A closer inspection of the experimental data in Figs. 3b and 3c indicates that both mechanisms described above are relevant. The two stems appear to be strongly coupled resulting in a small step upon splitting of the first stem into two stems at I_1 accompanied by a drop to about half in the penetration rate into each stem. On the other hand, instead of a symmetric splitting of the original stem at $y = 0$ into two stems at $y = \pm y_0$, the position of the first stem remains almost fixed and the second stem appears closely above it. This implies that even though the two stems are strongly coupled, their precise location is determined by the small local irregularities in the edge "coastline". Indeed, our lithographic process resulted in some edges roughness and in addition, the constriction edges have a shape of a rounded polygon rather than a semicircle (see SEM image in Fig. 1b). As a result, the third and fourth stems in Fig. 2o are seen to be formed at the rounded corners of the polygon. The fine details of the edge shape were incorporated in our TDGL simulations. Figures 5d and 5f clearly show enhanced suppression of the order parameter at these corners that become the nucleation points of new stems at higher currents as shown in Figs. 6a and 6b. The incorporated fine irregularities of the edges also result in the pronounced roughness of the order parameter at the left edge of the constriction where the current approaches J_d as seen in Fig. 5b and Movies 5 to 8, and lead to the differences in the vortex flow patterns between the upper and lower stems in Figs. 6a and 6b.

Supplementary References

1. Kramer, L. & Watts-Tobin, R. J. Theory of Dissipative Current-Carrying States in Superconducting Filaments. *Phys. Rev. Lett.* **40,** 1041–1044 (1978).
2. Ivlev, B. I. & Kopnin, N. B. Electric currents and resistive states in thin superconductors. *Adv. Phys.* **33,** 47–114 (1984).
3. Kopnin, N. *Theory of Nonequilibrium Superconductivity*. (Oxford University Press, 2001). doi:10.1093/acprof:oso/9780198507888.001.0001
4. Vodolazov, D. Y. & Peeters, F. M. Rearrangement of the vortex lattice due to instabilities of vortex flow. *Phys. Rev. B* **76,** 014521 (2007).
5. Ashcroft, N. W. & Mermin, D. *Solid State Physics*. (Harcourt College Publ., 1976).
6. Kogan, V. G. Pearl's vortex near the film edge. *Phys. Rev. B* **49,** 15874–15878 (1994).
7. Gurevich, A. & Mints, R. G. Self-heating in normal metals and superconductors. *Rev. Mod. Phys.* **59,** 941–999 (1987).
8. Bezuglyj, A. I. & Shklovskij, V. A. Effect of self-heating on flux flow instability in a superconductor near Tc. *Physica C* **202,** 234–242 (1992).
9. Van Sciver, S. W. *Helium Cryogenics*. (Springer, New York, 2012). doi:10.1007/978-1-4419-9979-5
10. Aranson, I., Gurevich, A. & Vinokur, V. Vortex Avalanches and Magnetic Flux Fragmentation in Superconductors. *Phys. Rev. Lett.* **87,** 067003 (2001).
11. Aranson, I. S. *et al.* Dendritic Flux Avalanches and Nonlocal Electrodynamics in Thin Superconducting Films. *Phys. Rev. Lett.* **94,** 037002 (2005).
12. Huebener, R. P. *Magnetic Flux Structures in Superconductors*. (Springer, Heidelberg, 2001). doi:10.1007/978-3-662-08446-5
13. Blatter, G., Feigel'man, M. V., Geshkenbein, V. B., Larkin, A. I. & Vinokur, V. M. Vortices in high-temperature superconductors. *Rev. Mod. Phys.* **66,** 1125–1388 (1994).
14. Zeldov, E., Clem, J. R., McElfresh, M. & Darwin, M. Magnetization and transport currents in thin superconducting films. *Phys. Rev. B* **49,** 9802–9822 (1994).
